# Exocycilic DNA Adducts in a Murine Model of Non-alcoholic Steatohepatitis

**DOI:** 10.4172/2157-2518.S3-003

**Published:** 2013-10-18

**Authors:** Marco E M Peluso, Armelle Munnia, Mirko Tarocchi, Mario Arciello, Clara Balsano, Roger W Giese, Andrea Galli

**Affiliations:** 1 Cancer Risk Factor Branch, Cancer Prevention and Research Institute, Florence, Italy; 2 Department of Experimental and Clinical Biomedical Sciences, University of Florence, Florence, Italy; 3 Department of Internal Medicine and Medical Specialties “Sapienza” University of Rome, Rome, Italy; 4 CNR-IBPM Istituto di Biologia e Patologia Molecolare, Rome, Italy; 5 Department of Pharmaceutical Sciences in the Bouve College of Health Sciences, Barnett Institute, Northeastern University, Boston, Massachusetts, USA

**Keywords:** C57BL/6 Mice, High-fat and cholesterol diet, NASH, Reactive oxygen species, Lipid peroxidation, MDA, Exocyclic DNA adducts, M_1_dG adducts

## Abstract

**Introduction:**

Non-alcoholic fatty liver disease is the most common hepatic disorder in Western countries. The transition from abnormal accumulation of lipids toward non-alcoholic steatohepatitis (NASH) represents a key step in the development of chronic liver pathologies. Oxidative stress and lipid peroxidation have often been proposed as mechanisms in the progression to steatohepatitis.

**Methods:**

We have examined the hepatic levels of exocyclic DNA adducts, indicated from 3-(2-deoxy-β-D-erythro-pentafuranosyl)pyrimido[1,2-α]purin-10(3H)-one deoxyguanosine (M_1_dG) adduct, a biomarker of oxidative stress and lipid peroxidation, in a murine model of NASH using the ^32^P-DNA postlabeling assay.

**Results:**

Our findings show that C57BL/6 mice fed with high-fat and cholesterol diet developed signs associated with NASH after eight weeks, whereas there was no evidence of steatosis in control mice. The score for steatohepatitis ranged from grade 2 to 3 for steatosis, inflammation, and fibrosis, showing that the experimental diet was able to induce pathologic alterations of the parenchyma in eight weeks. Higher levels of M_1_dG adducts were detected in the livers of C57BL/6 mice which developed experimental NASH after eight weeks of high-fat and cholesterol feed, 5.6 M _1_dG ± 0.4 (SE) per 10^6^ total nucleotides, as compared to control mice, 1.6 M_1_dG ± 0.4 (SE). The statistical analysis showed that the increment of oxidatively damaged DNA in mice with NASH raised on high-fat and cholesterol diet was statistically significant as compared to control mice, P=0.006.

**Conclusions:**

Our report suggests a link between NASH and M_1_dG in experimental animals fed with a diet rich in saturated fats and cholesterol. High-fat and cholesterol may act together in inducing a broader spectrum of oxidatively damaged DNA, including exocyclic DNA adducts, that may contribute to the decline of hepatocyte functions, from disturbance of critical pathways, such as transcription and replication, triggering transient or permanent cell-cycle arrest and cell-death, up to chromosomal instability.

## Introduction

Non-Alcoholic Fatty Liver Disease (NAFLD) is the most common hepatic disorder in Western countries affecting 15%−46% of adults. NAFLD is a condition characterized by excess fat in absence of substantial inflammation or fibrosis in the liver of people who drink little or no alcohol. NAFLD is emerging as the most important cause of chronic liver diseases due to increment in incidence of overweight, obesity and diabetes mellitus type 2 in general population. The transition from abnormal accumulation of lipids, particularly triglycerides within hepatocytes (steatosis), toward Non-Alcoholic Steatohepatitis (NASH) represents a key step in the development of chronic liver pathologies. Indeed, NASH may progress to advanced fibrosis, liver cirrhosis, and Hepatocellular Carcinoma (HCC) [1,2]. Recently, the rate of onset of HCC in patients with NASH has been reported to be comparable to that of the patients with chronic hepatitis C [2].

Contributing factors for NASH include combined features of Western lifestyle, such as diet rich in fatty acids and cholesterol [3]. Nevertheless, the molecular mechanisms that drive the progression from steatosis to NASH have not been completely elucidated. NASH is thought to develop via the “two-hit” hypothesis [4]. According to this model, simple steatosis is the critical “first hit”, which enhances the vulnerability of the liver to the factors that constitute the “second hit” that will promote hepatocyte injury, inflammation and fibrosis. Persistent oxidative stress and peroxidation of lipids (LPO) are the most popular mediators of the second hit in the “two-hit model. Accumulating experimental data indicate also that mitochondrial dysfunctions may play a role in the pathogenesis of NASH [5].

In particular, LPO produces malondialdehyde (MDA; β-hydroxyacrolein) [6]. MDA is also a by-product of prostaglandin biosynthesis. MDA is not only a product of physiological metabolism, but derives from oxidation of DNA, which generates base propenals, that are structural analogs of the enol tautomer of MDA [7]. As result, elevated levels of 3-(2-deoxy-β-D-erythro-pentafuranosyl) pyrimido [1,2-α]purin-10(3H)-one deoxyguanosine adduct, known as M_1_dG, are generated. Exocyclic DNA adducts, including M_1_dG, tend to induce base pair and frameshift mutations in repeated sequences [8]. Cline et al. showed that M_1_dG impairs the transcription of mitochondrial genes [9]. In healthy human tissues, M_1_dG occur at levels comparable to the most abundant form of base oxidation, e.g. 7,8-dihydro-8-oxo-2’-deoxyguanosine (8-oxodG). Moreover, M_1_dG adducts are considered a biomarker of environmental exposures [10,11] and dietary habits [12–15], predictive of cancer development and tumor progression [12,16–19]. Recently, we showed that M_1_dG is associated with aberrant methylation in the Long **Interspersed** Nuclear Element-1 repeated elements and in the promoter region of *interleukin-6* [20]. Our previous studies suggested also a link between inflammation, M_1_dG adducts and myeloperoxidase (MPO) catalysed production of hypochlorous acid, a main Reactive Oxygen Species (ROS), in the lung of C57B/6 mice [21,22].

In this report, we have examined the generation of exocyclic DNA adducts, indicated from M_1_dG, a biomarker of oxidative stress and LPO, in a murine model of NASH [23]. The hepatic levels of M_1_dG adducts have been measured using the ^32^P-DNA postlabeling assay [10,13], a highly sensitive technique widely employed for the analysis of oxidatively damaged DNA induced from ROS and LPO by-products [14,19,24,25]. In detail, we measured the amounts of M_1_dG adducts in the livers of C57BL/6 mice after eight weeks of a high-fat diet (42.0% kcal from fat) with added cholesterol (0.2%, w/w) in respect to control mice, thereby studying a dietary pattern which is often considered to mimic the Western diet [26].

## Material and Methods

### Animals and experimental design

A recent study has suggested that only mice on a diet rich in saturated fats and cholesterol developed fibrosing steatohepatitis, suggesting that both dietary cholesterol and dietary fat are necessary for the development of NASH [27]. Therefore, we aimed to explore some of the molecular mechanisms by which dietary fat and dietary cholesterol induce NASH development. In detail, starting at four weeks of age, twelve male C57BL/6 mice (Harlan Laboratories S.r.l. Udine, Italy) were randomly assigned to two groups receiving two different diets for eight weeks: (1) high-fat and cholesterol (HFC) diet, 42.0% kcal from fat (anhydrous milk fat), with 0.2% (w/w) of total cholesterol (0.15% added and 0.05% from fat source) (TD.88137, Harlan Laboratories S.r.l. Udine, Italy), and (2) standard chow, 17.0% kcal from fat (crude oil) (TD.2018, Harlan Laboratories S.r.l. Udine, Italy). The twelve mice were kept on a regular dark/light cycle, and received water and regular or HFC chow ad libitum. Dietary fat compositions and fatty acid profiles of the HFC diet have been previously reported [26]. After eight weeks of HFC diet feed, mice were weighed and the liver was harvested, weighed, and apportioned for DNA extraction as flash-frozen tissue or preserved in 10% buffered formalin. Animal procedures were performed in accordance with the guidelines of the General Hospital Institutional Committee that reviewed and approved the protocol.

### Histopathology

Formalin-preserved liver tissue samples were embedded in paraffin and sectioned (5 μm thick). Deparaffinized, hydrated serial liver tissue sections were stained with haematoxylin-eosin and picrosirius red using standardized protocols of the Department of Experimental and Clinical Biomedical Sciences of the University of Florence [28,29]. Tissue sections stained with haematoxylin-eosin were analysed for common symptoms of NASH by a hepatologist who was blinded to the study. The classification of Brunt et al. [30] was used to assign numerical scores to steatosis, inflammation and fibrosis.

### Reference adduct standard

A reference adduct standard was prepared: calf-thymus (CT)-DNA was treated with 10 mM MDA (ICN Biomedicals, Irvine, CA, USA), as previously reported [31]. MDA treated CT-DNA was diluted with untreated DNA to obtain decreasing levels of the reference adduct standard to generate a calibration curve.

### DNA extraction and purification

DNA was extracted and purified from the livers of C57BL/6 mice using a method that requires digestion with ribonuclease A, ribonuclease T1 and proteinase K treatment and extraction with saturated phenol, phenol/chloroform/isoamyl alcohol (25:24:1), chloroform/isoamyl alcohol (24:1) and ethanol precipitation, as previously described [32,33]. DNA concentration and purity were determined using a spectrophotometer. Liver DNA samples were subsequently stored at −80°C.

### Mass spectrometry

DNA adducts in MDA treated CT-DNA sample were analyzed by mass spectrometry (Voyager DE STR from Applied Biosystems, Framingham, MA), as reported elsewhere [34,35], through the following sequence of steps: (1) reaction of DNA with NaBH_4_ followed by precipitation with isopropanol [36]; (2) digestion with snake venom phosphodiesterase and nuclease P1; (3) extraction of DNA adducts that are less polar than normal nucleotides on an OASIS cartridge (Waters Corp.); (4) tagging with an isotopologue pair of benzoylhistamines (d_o_ and d_4_) in a phosphate-specific labeling reaction in the presence of carbodiimide; (5) removal of residual reagents by ion exchange solid-phase extraction; (6) resolution of tagged adducts by capillary reversed-phase HPLC with a collection of drops onto a MALDI plate; (7) addition of matrix (α-cyano-4-hydroxycinnamic acid); and (8) analysis by matrix-assisted laser desorption/ionization time-of-flight mass spectrometry (MALDI-TOF-MS).

### ^32^P-DNA postlabeling assay

The hepatic levels of exocyclic DNA adducts, indicated from M_1_dG, a biomarker of oxidative stress and LPO, were measured using a modified version of the ^32^P-DNA postlabeling assay [10,37]. In detail, DNA (2 μg) was hydrolyzed by incubation with micrococcal nuclease (21.45 mU/μl) and spleen phosphodiesterase (6.0 mU/μl) at 37°C for 4.5 h. Hydrolyzed DNA was treated with nuclease P1 (0.1 U/μl) at 37°C for 30 min [38]. After enzymatic treatment, samples were incubated with 25 μCi of carrier-free [γ−^32^P] ATP (3000 Ci/mM) and polynucleotide kinase T4 (0.75 U/μl) to generate ^32^P-labeled adducts at 37°C for 30 min [38]. ^32^P-labeled adducts were applied on polyethyleneimine cellulose thin-layer chromatography plates (Macherey-Nagel, Germany) and processed as previously described [13]. This chromatographic modification of the ^32^P-postlabeling method has been developed from our laboratory for the specific detection of this specific kind of exocyclic DNA adducts [13] by using a low-urea solvent system known to be effective for the detection of low molecular weight and highly polar DNA adducts. In brief, ^32^P-labeled products were applied to the origin of chromatograms and developed with 0.35 MgCl_2_ up to 2.0 cm filter paper wick. Plates were developed in the opposite direction with 2.1 M lithium formate, 3.75 M urea, pH 3.75, and then run at the right angle to the previous development with 0.24 M sodium phosphate, 2.4 M urea, pH 6.4.

Then, the detection and quantification of M_1_dG adducts and total nucleotides (nt), i.e. diluted samples that were not treated with NP1, were performed by storage phosphor imaging techniques employing intensifying screens from Molecular Dynamics (Sunnyvale, CA, USA). The intensifying screens were scanned using a Typhoon 9210 (Amersham). Software used to process the data was ImageQuant (version 5.0) from Molecular Dynamics. After appropriate background subtraction, the levels of M_1_dG adducts were expressed such as Relative Adduct Labelling (RAL)=pixels in adducted nucleotides/pixels in nt. The levels of M_1_dG adducts were corrected across experiments based on the recovery of reference standard. Co-chromatography of the liver DNA samples together with the MDA treated DNA adduct standard was performed to identify the M_1_dG adducts detected in the chromatograms of experimental animals using 2.1 M lithium formate, 3.75 M urea, pH 3.75 and 0.24 M sodium phosphate, 2.4 M urea, pH 6.4 or 0.24 M sodium phosphate, 2.7 M urea, pH 6.4.

### Statistical analysis

Animal data are expressed as average ± SD. The levels of M_1_dG adducts were log-transformed to normalize the distribution and stabilize the variance. Student’s t-test was calculated to compare experimental groups. P<0.05 (two-tailed) was considered significant. Data were analyzed using SPSS 13.0 (SPSS, USA).

## Results

### Animals and high-fat and cholesterol diet

**Figure 1:**
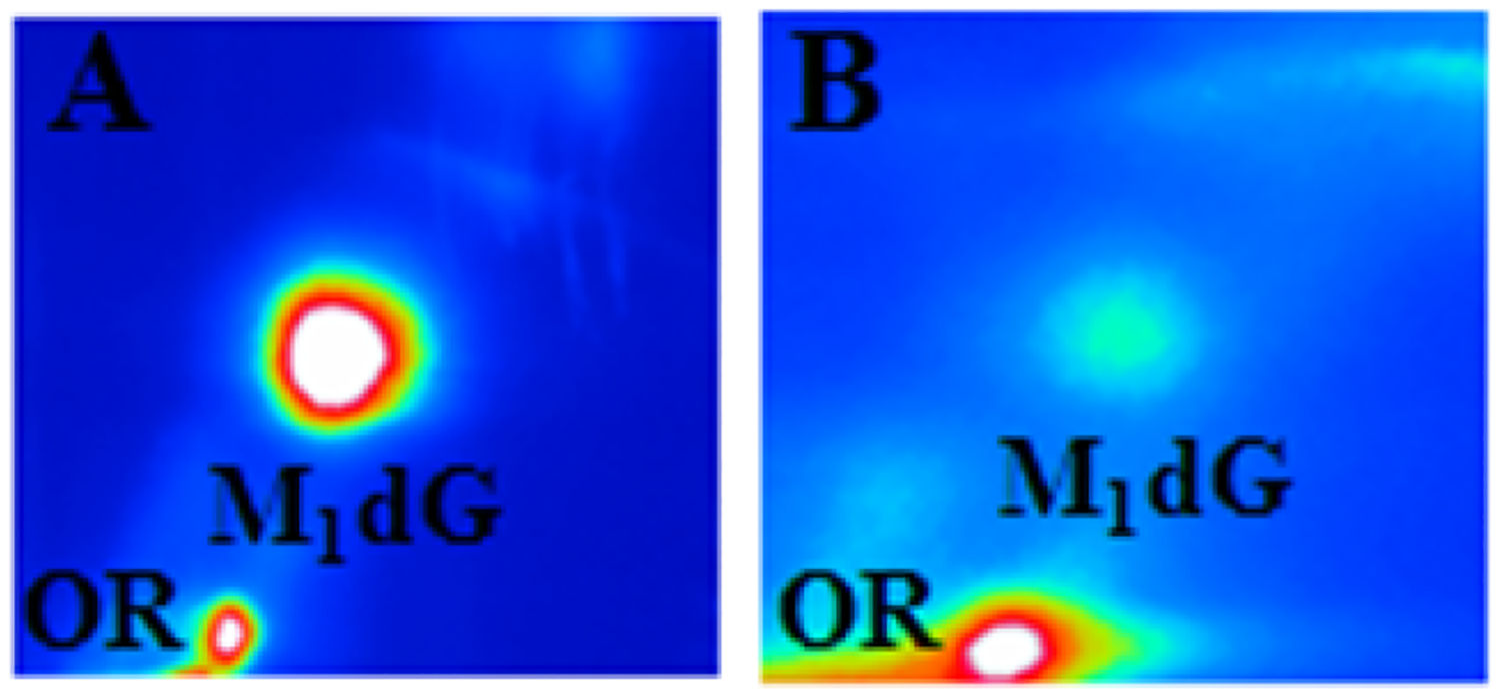
(A) Representative images of liver sections stained with hematoxylin and eosin or sirius red staining after eight weeks of normal or high-fat and cholesterol diet chow. (B) Total animal weight, liver weight and relative ratio (liver weight/total weight) express in grams as average ± SD.

Hematoxylin-eosin-stained sections of liver tissue were scored for signs of NASH by a pathologist blinded to the study. Table 1 shows the scores for steatosis, inflammation, and fibrosis that were assigned according to the classification of Brunt et al. [30]. HFC diet fed mice developed signs associated with NASH after eight weeks, conversely, there was no evidence of steatosis in control mice (Figure 1A). The score for steatohepatitis for HFC diet fed mice ranged from grade 2 to 3 for steatosis, inflammation, and fibrosis, showing that the HFC diet was able to induce a pathologic alteration of the parenchyma in eight weeks. HFC diet fed mice were significantly more overweight, on average of 44 g ± 3.3 g than mice with standard chow (34 g ± 1.2 g). HFC diet induced a significant enlargement of liver representing 7.2% of total weight compared to 4.8% for control animals (Figure 1B).

### Reference adduct standard by ^32^P-postlabeling and mass spectrometry

There were 5.0 M_1_dG adducts ± 0.6 per 10^6^ nt in MDA-treated CTDNA based on ^32^P-postlabeling. The presence of the M_1_dG adduct in this sample was confirmed by MALDI-TOF-MS as reported before [34,35], and we are using the nomenclature reported by Goda and Marnett for this adduct [36]. A calibration curve was then set up by diluting this sample with untreated CT-DNA and measuring the decreasing level of M_1_dG, r-squared=0.99.

### M_1_dG adducts in experimental animals

**Figure 2:**
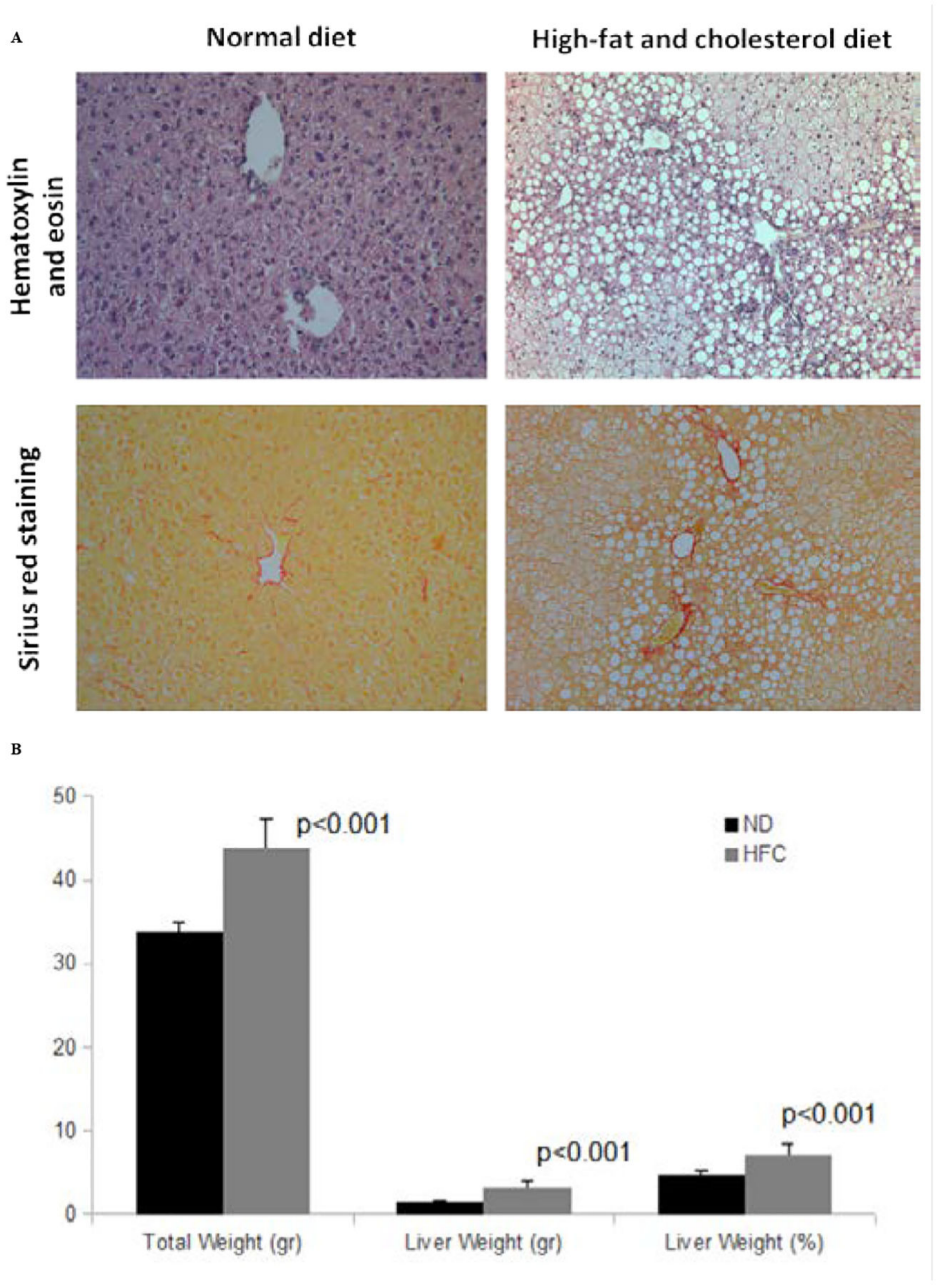
The pattern of the 3-(2-deoxy-β-D-erythro-pentafuranosyl) pyrimido[1,2-α]purin-10(3H)-one deoxyguanosine adduct-spot in the liver of a C57BL/6 mice, that developed an experimental non-alcoholic steatohepatitis after eight weeks of high-fat and cholesterol diet feed (A) and in the liver of a control mice (B).

**Figure 3:**
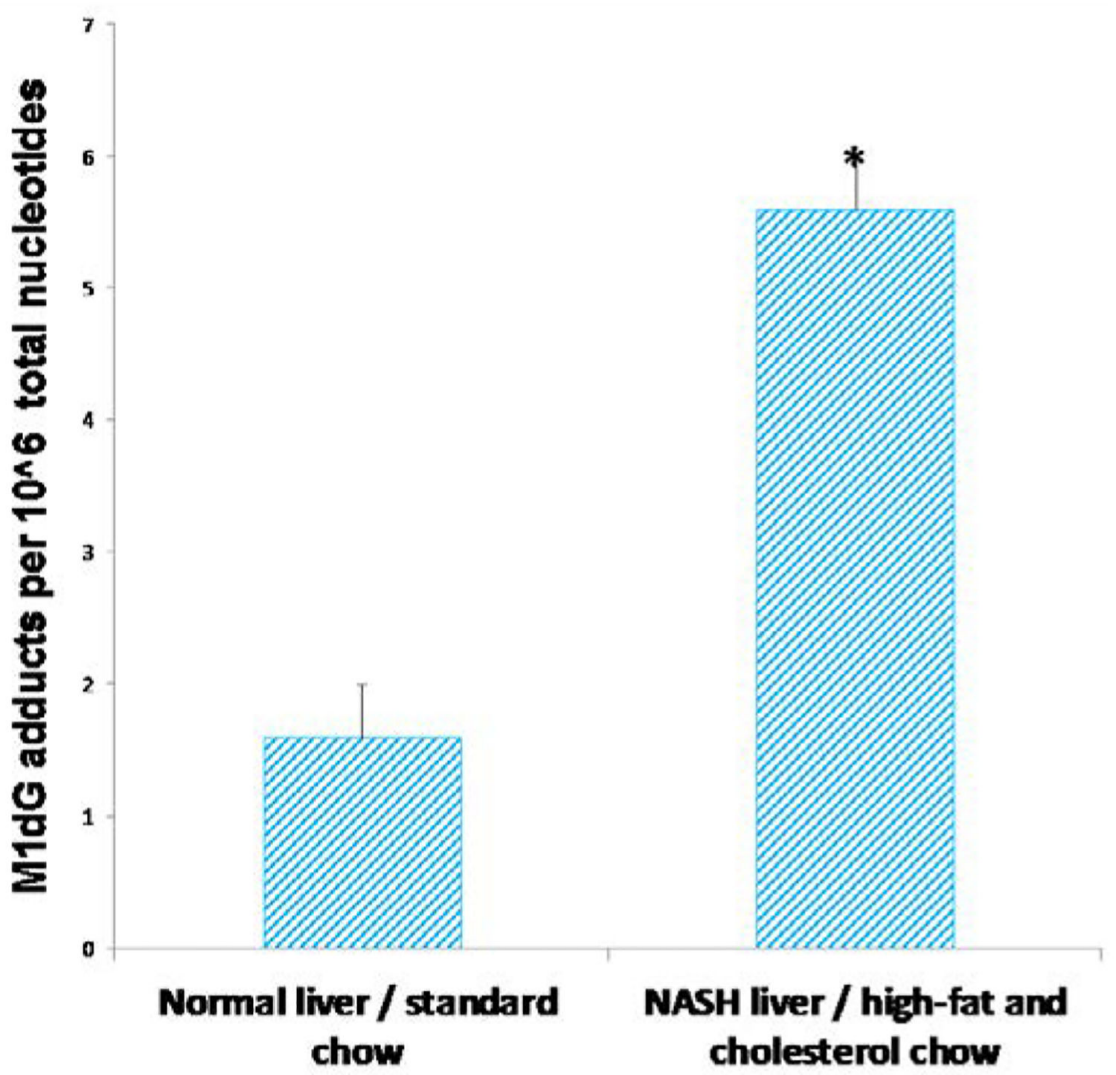
Mean levels of the 3-(2-deoxy-β-D-erythro-pentafuranosyl) pyrimido[1,2-α]purin-10(3H)-one deoxyguanosine adduct in the livers of six male C57BL/6 mice, that developed an experimental non-alcoholic steatohepatitis after eight weeks of high-fat and cholesterol diet feed in respect to six control mice. P=0.006 vs. control animals.

A typical pattern of M_1_dG adduct spot was detected in the chromatograms of C57BL/6 mice. As expected, the intensity of M_1_dG adduct spots was stronger in the chromatograms of mice, which developed experimental NASH after eight weeks of HFC feed (Figure 2A), as compared to mice without NASH (Figure 2B). Co-chromatography confirmed the presence of M_1_dG adducts in the liver DNA of experimental animals. The mean levels of M_1_dG adducts were 5.6 adducts ± 0.4 (SE) per 10^6^ nt in the livers of six mice with NASH and HFC diet and 1.6 adducts ± 0.4 (SE) per 10^6^ nt in normal non-steatotic livers of six control mice without NASH (Figure 3). Subsequently, the result of the statistical analysis showed that the levels of a specific type of exocyclic DNA adducts in the livers of mice with NASH and HFC diet was statistically significantly different from those of mice without NASH and with a control chow, P=0.006.

## Discussion

In the present study, we have examined the hepatic levels of exocyclic DNA adducts, indicated from M_1_dG adducts, a biomarker of oxidative stress and LPO, in a murine model of NASH. The liver of C57BL/6 mice, a tissue sensitive to ROS induced by a range of agents, was used as a model system of NASH pathogenesis [23]. A recent study of Savard, et al. [27] have suggested that dietary fat and dietary cholesterol interact in the development of the hepatic histological abnormalities of NASH. In the present study, we observed a link between NASH and the generation of oxdatively damaged DNA in the livers of experimental rodents fed with HFC diet. The levels of M_1_dG adducts were statistically significantly increased in the livers of mice which developed experimental NASH after eight weeks of HFC feed in respect to control mice raised on a standard chow. High levels of a specific type of exocyclic DNA adducts were detected in mice with high score for hepatic histological abnormalities of steatohepatitis. High-fat diet and cholesterol may act together in inducing NASH by increasing the intracellular amounts of oxidative stress within hepatocytes, and, consequently, causing oxidation of DNA and LPO, which generate biological aldehydes, including MDA and base propenals, measured as M_1_dG adducts. Since NASH is an inflammatory condition, other types of exocyclic DNA adducts induced from LPO derived aldehydes are expected, such as those generated by 4-hydroxy-2’-nonenal and acrolein. For a complete story of the relationship between inflammation and DNA damage in NASH, our data support a disease mechanism that includes a broader spectrum of oxidatively damaged DNA, including 8-oxodG.

Many studies have analyzed the association between high-fat diets with biomarkers of oxidative stress and LPO in experimental animals and humans. For an example, Chakravarthy et al. examined the effects of HFC diet, which is also used in the present study, on LPO biomarkers. A significant association with high levels of urinary 15-isoprostane F2t was found in mixed BL/6 and 129 mice [26]. Dimitrova-Shumkovska et al. demonstrated that HFC diet causes a significant enhancement of LPO, measured such as thiobarbituric acid reactive substances, in livers of Wistar rats [39]. A molecular epidemiology study conducted by Leuratti et al. showed that the frequent consumption of saturated fats is significantly associated to high levels of colorectal exocyclic DNA adducts within the participants of the United Kingdom Flexible Sigmoidoscopy and the European Prospective Investigation on Cancer studies [12]. The relationship between high-fat diets with endogenous damage was recently analysed from Moore et al. in a randomized controlled intervention study [40]. In that study, the levels of M_1_dG adducts were found to be significantly increased in peripheral blood cells of volunteers on the high saturated fat diet in two weeks. High-fat diet may increase the levels of oxidative stress and the production of reactive aldehydes in different manners, including by causing saturation of β-oxidation pathway in mitochondria [41], by altering expression of genes involved in ROS generation and free radical scavenger [42], and by inducing inflammation [21,22]. An attractive hypothesis is that cholesterol may increase the intracellular levels of oxidative stress within hepatocytes by activating Kupffer cells [43], macrophages with strong ROS production capacity, and by enhancing LPO susceptibility [44]. Indeed, the attack of ROS on the 5,6-double bond and the concomitant vinylic methylene group at C-7 in the B ring of cholesterol may generate peroxyl radicals [45]. A pro-oxidant role for dietary cholesterol has been also suggested from the study of Cazzola et al. [46], which showed that hypercholesterolemia increased LPO susceptibility in overweight and obese subjects.

The molecular mechanisms that drive the progression from steatosis to NASH are unclear. Our study shows that mice fed with HFC diet for eight weeks develop histological changes in their livers with symptoms of steatosis, inflammation and fibrosis. These changes were accompanied by a significant increase in the levels of M_1_dG in liver DNA, a highly pro-mutagenic lesion which may contribute to transition to NASH. The presence of high levels of M_1_dG adducts in NASH livers supports the involvement of oxidative stress and redox imbalance in NASH aetiology. In this way, the disturbance of the normal redox state of hepatocytes can promote the formation of a vicious cycle of ROS production. Excess ROS may exert their detrimental effects on hepatocytes via attack on DNA and inner membrane lipids causing increased generation of exocyclic DNA adducts and LPO. Persistent DNA damage may, then, contribute to the decline of hepatocyte functions, by disturbance of critical pathways, such as transcription and replication, and by causing transient or permanent cell-cycle arrest and cell-death, up to inducing chromosomal instability. One could argue that if the continuous production of ROS and oxidatively damaged DNA persists in steatotic liver, the risk of transition from abnormal accumulation of lipids toward NASH up to other chronic liver diseases, such as cirrhosis and hepatocellular carcinoma, increases.

Our findings join a growing body of experimental studies, which examined the levels of biomarkers of oxidative stress in NASH livers in respect to normal non-steatotic livers. In particular, the pattern of DNA damage observed in present study reflects that reported from Seki et al., which examined the relationship between the levels of 8-oxodG and 4-hydroxy-2’-nonenal, two biomarkers of oxidatively damage DNA and LPO, in the livers of patients with NASH [47]. In that study, the biomarkers of oxidative stress were significantly increased in the livers with NASH as compared to normal non-steatotic livers. Fujita et al. analyzed the levels of 8-oxodG in the liver of patients with NASH in respect to healthy controls [48]. In that study, the levels of oxidatively damage DNA, were significantly increased in the livers of subjects with NASH. High levels of hepatic MDA and 8-oxodG were recently observed in the steatotic liver of C57BL/6 mice fed with HFC diet in respect to control mice [49], suggesting that the continuous attack of ROS against DNA plays a role in the progression of liver disorders.

M_1_dG is repaired in cells by the Nucleotide Excision Repair (NER) system [50], therefore, a limitation of the present study is the lack of measurements of hepatic NER capacity in experimental animals. Our study of DNA damage in the progression toward NASH would have been enhanced by an analysis of NER status in each animal cohort. The relationship between the expression of five NER genes and the NASH status was analyzed by Schults et al. [51]. In that study, only a modest decrement in the expression of the excision repair cross-complementing rodent repair deficiency, complementation group 4 gene was observed in NASH in respect to steatotic livers after stratification for MPO-immunoreactivity, a marker of neutrophil activity. Schults et al. [51] also showed that the hepatic levels of M_1_dG were not dependent on MPO release, in discrepancy with that previously observed in the lung of experimental animals with acute lung inflammation [22]. Some enzyme expected to be important in hepatic toxicity based on correlations from other organs, may be less relevant in the development of liver toxicity. Changes in hepatic MPO release may be not sufficient to modify the effects of the vicious cycle of ROS production in steatotic and NASH livers.

M_1_dG adduct is a form of DNA damage that is quite persistent, with a relatively long half-life of 12.5 days [6]. If unrepaired, M_1_dG adducts may alter cellular homeostasis by inhibiting transcription at DNA damage sites, and increase the risk of mutations in relevant genes. Persistent DNA damage would also result in strand breaks and direct initiation of cell death. The chemical properties of this kind of damage make M_1_dG also interesting with respect to its potential detrimental effects on mitochondrial gene expression and mitochondrial DNA integrity [9]. As no DNA repair mechanisms for M_1_dG adducts have been identified so far in mitochondria, it is plausible that this type of exocyclic DNA adducts may participate to the transition toward NASH also by causing dysfunctions in the mitochondria, such as decreased activity of respiratory chain and impaired mitochondrial β-oxidation.

A strength of our study is that the generation of M_1_dG adducts was measured using the ^32^P-postlabeling, a technique known to be sensitive for the detection of a wide range of carcinogens [25,37,52], including dietary carcinogens [14]. A high repeatability of DNA adduct measurements has been also shown for this assay [37,53]. The sensitivity of the ^32^P-postlabeling technique has been greatly enhanced by introducing the incubation of DNA digest with the Penicillium citrinum nuclease P1 before the ^32^P-labeling step [54]. This enrichment increased the sensitivity of the technique up to 1 adduct in about 10^9^-10^10^ nt. The nuclease P1 enrichment procedure was found to be applicable to the detection of aromatic or bulky non-aromatic DNA adducts formed with structurally different carcinogens, including MDA, benzoa(a)pyrene, 7,12-dimethyl-ben(a)anthracene, dibenzo(c,g) carbazole, 4-aminobiphenyl, safrole, and mitomycin C [17,18,38,54]. Nevertheless, the ^32^P-postlabeling is an assay that is unable to determine the structure of the adducts under exams; higher specificity may be obtained if the technique is coupled with the use of appropriate internal standards [14,19,31,37,55,56], or mass spectrometry [14,19,31,57,58], such as in the case of M_1_dG adducts [14,19,31].

## Conclusions

Our study shows that high levels of a specific type of exocyclic DNA adducts, the M_1_dG adducts, were detected in mice with high score for hepatic histological abnormalities of NASH that were fed with HFC diet in respect to control mice. Our findings broaden knowledge about the importance of dietary fats and cholesterol in the development of steatohepatitis, through the generation of oxidatively damaged DNA. High-fat diet and dietary cholesterol may act together by increasing the intracellular amounts of oxidative stress and ROS within hepatocytes. A continuous attack to DNA may contribute to the general decline of hepatocyte functions, from disturbance of DNA metabolism, such as transcription and replication, triggering transient or permanent cell-cycle arrest and apoptosis, up to genome instability.

## Figures and Tables

**Table 1:** Score for steatohepatitis including grading of steatosis, inflammation, and fibrosis in standard chow control mice and in mice with non-alcoholic steatohepatitis **(**NASH) fed with high-fat and cholesterol diet.

	N	Standard diet fed mice without NASH	N	High-fat and cholesterol diet fed mice with NASH
Steatosis (Grade)	6	0	6	3
Fibrosis (Grade)	6	0	6	3
Inflammation (Grade)	6	0	6	2–3

## Abbreviations:

HCC: Hepatocellular Carcinoma
HFC: High-Fat and Cholesterol
LPO: Lipid Peroxidation
MALDI-TOF-MS: Matrix-Assisted Laser Desorption/Ionization Time-Of-Flight Mass Spectrometry
MDA: Malondialdehyde
M_1_dG: 3-(2-deoxy-β-D-erythro-pentafuranosyl)pyrimido[1,2-α]purin-10(3H)-one
MPO: Myeloperoxidase
NAFLD: Nonalcoholic Fatty Liver Disease
NASH: Nonalcoholic Steatohepatitis
8-oxodG,7,8-dihydro-8-oxo-2’-deoxyguanosine
ROS: Reactive Oxygen Species
nt: Total Nucleotides
